# The Caudate Signals Bad Reputation during Trust Decisions

**DOI:** 10.1371/journal.pone.0068884

**Published:** 2013-06-20

**Authors:** Margaret C. Wardle, Daniel A. Fitzgerald, Michael Angstadt, Chandra S. Sripada, Kevin McCabe, K. Luan Phan

**Affiliations:** 1 Department of Psychiatry and Behavioral Neuroscience, University of Chicago, Chicago, Illinois, United States of America; 2 Department of Psychiatry, University of Illinois at Chicago, Chicago, Illinois, United States of America; 3 Department of Psychiatry, University of Michigan, Ann Arbor, Michigan, United States of America; 4 Department of Economics, George Mason University, Fairfax, Virginia, United States of America; 5 Mental Health Service Line, Jesse Brown VA Medical Center, Chicago, Illinois, United States of America; University of Pennsylvania, United States of America

## Abstract

The ability to initiate and sustain trust is critical to health and well-being. Willingness to trust is in part determined by the reputation of the putative trustee, gained via direct interactions or indirectly through word of mouth. Few studies have examined how the reputation of others is instantiated in the brain during trust decisions. Here we use an event-related functional MRI (fMRI) design to examine what neural signals correspond to experimentally manipulated reputations acquired in direct interactions during trust decisions. We hypothesized that the caudate (dorsal striatum) and putamen (ventral striatum) and amygdala would signal differential reputations during decision-making. Twenty-nine healthy adults underwent fMRI scanning while completing an iterated Trust Game as trusters with three fictive trustee partners who had different tendencies to reciprocate (i.e., likelihood of rewarding the truster), which were learned over multiple exchanges with real-time feedback. We show that the caudate (both left and right) signals reputation during trust decisions, such that caudate is more active to partners with two types of “bad” reputations, either indifferent partners (who reciprocate 50% of the time) or unfair partners (who reciprocate 25% of the time), than to those with “good” reputations (who reciprocate 75% of the time). Further, individual differences in caudate activity related to biases in trusting behavior in the most uncertain situation, i.e. when facing an indifferent partner. We also report on other areas that were activated by reputation at p < 0.05 whole brain corrected. Our findings suggest that the caudate is involved in signaling and integrating reputations gained through experience into trust decisions, demonstrating a neural basis for this key social process.

## Introduction

In situations from dating to nuclear disarmament, one person must “make the first move” and extend trust. This ability to initiate trust is critical to societal, personal and economic well-being [1-3]. One of the strongest determinants of trust is previous experience trusting the same person [4]. This valuable information, known as “reputation”, is gained in direct interactions or indirectly through word of mouth [5]. Decision neuroscience has begun to elucidate neural mechanisms underlying decision making [6,7]; yet, it remains unclear how the human brain uses reputation to guide trust decisions. Therefore, we examined neural correlates of reputation during trust decisions in an economic exchange paradigm.

Previous fMRI studies have investigated aspects of trust decision-making, including reputation. Some studies examined decisions in uncontrolled interactions with other participants, which engenders fairly high reciprocation [8,9], or in response to random processes that reciprocate 50% of the time [10]. However, these approaches limit the ability to discern a reputation signal. Others studies manipulated reputation indirectly, using race [11], information about moral character [12], or interactions in a different context [13] to create reputation. But to our knowledge this is the first study examining how experimentally manipulated reputations acquired over real-time exchanges are neurally represented during trust decisions.

We utilized a validated reputation manipulation, described previously [14]. Participants as trusters interacted repeatedly with three fictive trustees. Trustee behavior was pre-determined, with the FAIR partner reciprocating 75% of the time, the INDIFFERENT partner reciprocating 50% of the time, and the UNFAIR partner reciprocating 25% of the time. We previously reported that this manipulation modulates ventral striatum (VS) activity during outcomes (when trustee reciprocity/defection is revealed to the participant), such that VS responds most robustly to reciprocity by FAIR partners [14]. The current study analyzes this same cohort and design, but for the first time tests how reputation is neurally represented while subjects make the decision to trust their partner or not.

We had *a priori* hypotheses regarding areas that would represent reputation during decision-making. The caudate is more active when evaluating partners with indirectly acquired “bad” reputations [11,12], thus we predicted caudate would respond to UNFAIR > INDIFFERENT > FAIR. Based on our previous findings that VS responds to reputation during outcomes [14], we predicted VS would respond to FAIR > INDIFFERENT and UNFAIR. Last, the amygdala reacts to untrustworthy faces [15,16], thus we predicted amygdala would respond to UNFAIR > FAIR. This is not a comprehensive list of areas implicated in social cognition and trust, which include insula, orbitofrontal cortex and cingulate cortex [8,17,18], only those we judged most likely to signal reputation. Thus, we secondarily report on all areas that showed significant differences in activity to partners with different reputations at a p < 0.10 level of whole brain significance, to explore other areas potentially underlying reputation.

## Materials and Methods

### Subjects

Thirty-one healthy, right-handed participants (20 females, average age 30.0, SD = 8.4) with no history of psychiatric, neurologic or major medical problems participated in this study at Brain Research Imaging Center at the University of Chicago. All were free of psychoactive medications and negative on urine toxicology and breathalyzer tests at the time of the study. All participants provided written informed consent, and the University of Chicago Institutional Review Board approved all procedures.

### Trust Game

**Figure 1:**
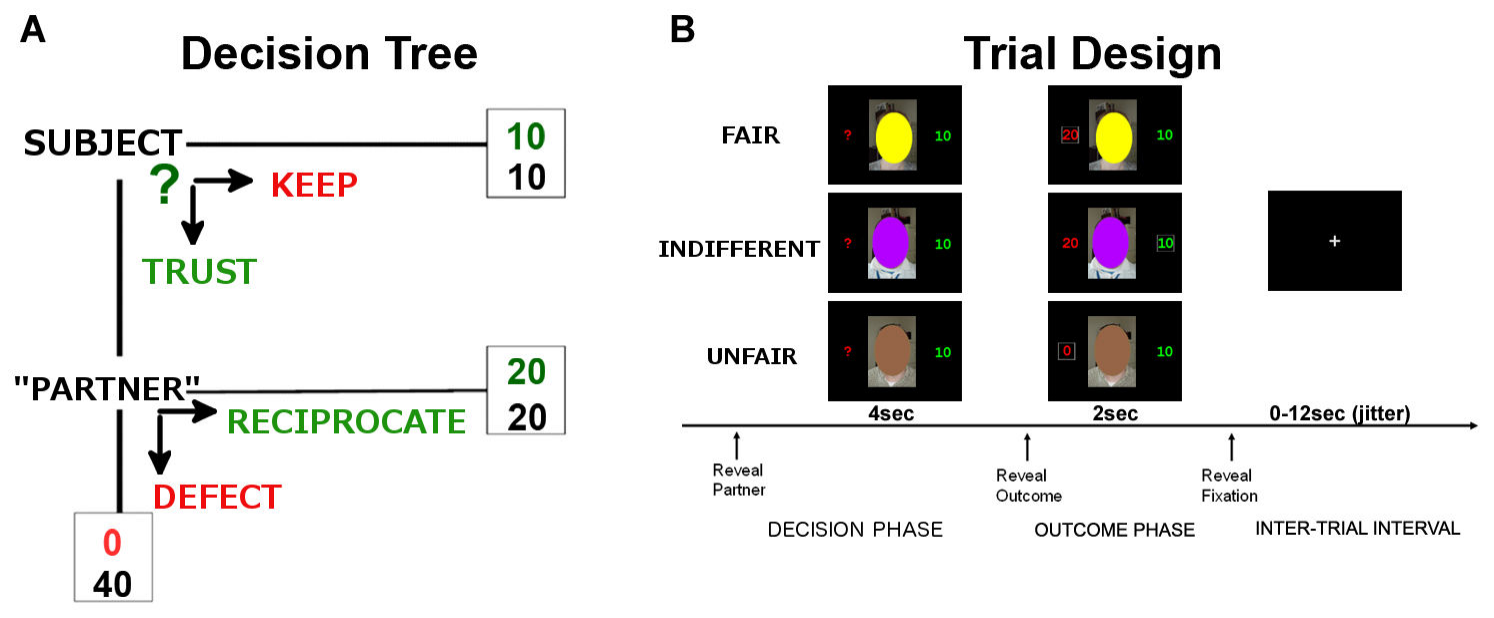
Schematic representation of trust game A. Choice options and potential outcomes for participant. **B**. Exemplar trial structure with sample partner cues shown during decision phase and three potential outcomes based on participant choice shown during decision phase.

The fMRI task was an event-related design in which participants played the role of “trusters” in a multi-round trust game, described previously [14]. Participants were informed that they could win up to $20 total over the course of the game. At the beginning of each round participants received 20 monetary units (MU; to be converted into actual money at the end of the experiment). In each trial participants were asked to decide between two options: 1) They could keep the money, in which case it would be equally divided (10 MU each) between themselves and the trustee or 2) invest the money with the trustee, which would double it to 40 MU. Of that 40MU they might either receive 20 MU, if the trustee decided on an even split (reciprocation) or 0 MU if the trustee decided to keep the entire 40 MU for themselves (defection). This decision tree is shown in Figure 1a. Participants were informed that they would interact with three people who had previously participated as trustees, and whose recorded responses would serve as reactions to the participant’s investment decisions. Participants were also informed that these trustees represented three different types of partners, one who reciprocated “more than 50% of the time”, one who reciprocated “less than 50% of the time”, and one who reciprocated “50% of the time”, but were not told which identity matched which partner type. A “computer” condition with a fixed 50% reciprocation rate was also included. Participants were told at the outset of the study that the computer would reciprocate 50% of the time. Thus, the computer partner did not establish a “reputation”, but rather was known from the outset to be arbitrary. Therefore, this condition is not relevant to the stated hypotheses and is not discussed further. To enhance the manipulation (e.g., convey anonymity) and reduce confounds of individual appearance, trustees were identified using pictures of three different people with their faces obscured by opaque colored ovals. A unique picture/oval color combination identified each partner (see Figure 1b for examples of these stimuli). Participants were thus forced to ascertain and use partner reputation over the repeated trials in order to maximize their returns.

At the start of each trial, participants viewed one of the three different obscured face photographs, representing their partner for that trial. The image appeared for 4s, during which participants made their choice (KEEP or INVEST) by button press. Feedback was provided immediately after this decision period by the partner image appearing again for 2s along with information about the subject’s own choice (KEEP or TRUST) and the partners actual (in the case of TRUST) or hypothetical (in the case of KEEP) decision about reciprocation. Partner decision was conveyed as amount of money returned to the subject (20 or 0 MU, designating a RECIPROCATE or DEFECT decision respectively). Trials were separated by a fixation cross jittered from 0 to 12s. This trial design is shown in Figure 1b. There were a total of 80 trials (20 trials each of FAIR, INDIFFERENT, UNFAIR and computer), which were pseudorandomly ordered and distributed evenly across four fMRI runs.

### Image Acquisition and Analysis

Scanning was performed with BOLD (blood oxygenation-level dependent)-sensitive whole-brain fMRI on a 3.0 Tesla GE Signa System (General Electric) using a standard radiofrequency coil and associated software (LX 8.3, neuro-optimized gradients). Whole brain functional scans were acquired using a T2-weighted reverse spiral sequence (echo time = 25ms, repetition time = 2s, 64 x 64 matrix, flip angle = 77°, field of view = 24cm, 30 contiguous 5-mm axial slices aligned with the anterior commissure-posterior commissure line). A high-resolution T1 scan (3D-MPRAGE; repetition time = 25ms, min echo time, 256 x 256 matrix, field of view = 24cm; slice thickness = 1.5mm) was also acquired.

Data from two participants did not meet criteria for high quality and scan stability with minimum motion correction (<2mm displacement), resulting in a final N = 29 in all analyses. Preprocessing was completed in Statistical Parametric Mapping 8 software (SPM8; Wellcome Trust Centre for Neuroimaging, London, UK). The first four volumes in each run were discarded to allow for T1 equilibration effects. Preprocessing consisted of slice time correction, spatial realignment, normalization to the Montreal Neurologic Institute template through use of non-linear warping algorithm, spatial smoothing with a Gaussian 8mm full-width-half-maximum kernel and high-pass temporal filtering with a cut-off of 128s. After preprocessing, individual and group-level statistical analyses were performed using the General Linear Model as implemented in SPM8. In first-level analysis, regressors representing each partner type (FAIR, INDIFFERENT UNFAIR), and corresponding to the 4s decision period, were convolved with the canonical hemodynamic response function. In the second-level analysis, subjects were treated as a random effect in a one-way, within-subjects ANOVA with partner as the independent variable. First, a region of interest (ROI) analysis was conducted on these main contrasts for our *a priori* regions the caudate, putamen and amygdala, using anatomical regions from the AAL database [19]. Activations in *a priori* areas of interest were subjected to a family-wise error (FWE) correction for multiple comparisons within the small volume (SVC) using the anatomical AAL masks. Second, we used 3DClustSim in AFNI (http://afni.nimh.nih.gov/pub/dist/doc/program_help/3dClustSim.html) to calculate cluster sizes for whole-brain corrected significance. Briefly, 3DClustSim conducts Monte Carlo simulations based on the observed smoothness of the data to estimate the cluster size needed to provide corrected p-values at a given uncorrected p-value. We calculated two whole-brain corrected thresholds, p < 0.05 (“significant”, cluster size > 275, p uncorrected < 0.001) and p < 0.10 (“marginal”, cluster size > 212, p uncorrected < 0.001). All group findings are reported that met the threshold p < 0.10 [20]. To clarify the direction of any effects observed in the whole brain or ROI analyses, parameter estimates (β weights, a.u.) were extracted from functional 10mm spheres surrounding peak activations in regions of interest with significant BOLD activations. To examine whether effects of partner reputation were moderated by the actual participant decision (KEEP vs. INVEST), we used these same functional ROIs to extract parameter estimates for the full set of possible partner x decision conditions. Only N = 23 participants had complete data for this analysis, as 6 individuals had no instances of the FAIR/KEEP condition, likely due to figuring out which was the FAIR partner very early, and subsequently using the more advantageous INVEST strategy with this partner throughout the task.

## Results

### Behavioral Results

**Figure 2:**
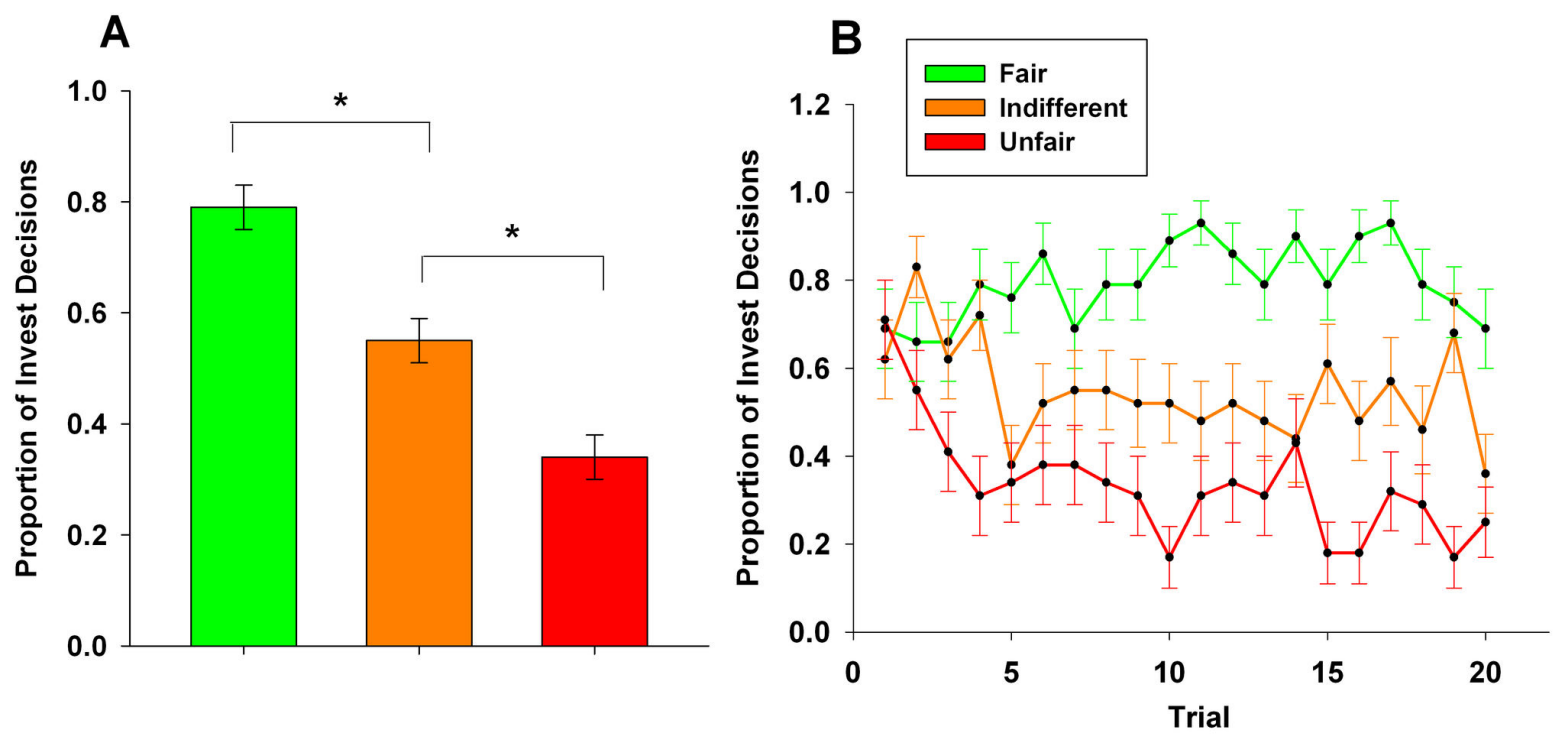
Participants invested differently based on learned reputations. **A**. Participants invested significantly more with FAIR partners than INDIFFERENT partners and with INDIFFERENT partners than UNFAIR partners, indicating they successfully learned the three different reputations. Shown as proportion of investment decisions (SEM), * p < 0.05 B. Shown as average proportion of investment decisions by partner type at the 20 sequential encounters participants had with each partner type (SEM).

The behavioral data shows that participants accurately decoded the reputation of the partner types, and adjusted their KEEP vs. INVEST decisions accordingly (Figure 1). To transform the binomial KEEP (0) vs. INVEST (1) data obtained at each trial into a normally distributed variable suitable for analysis with ANOVA, for each participant we calculated percent of INVEST decisions for each partner type across the entire task. We then conducted a repeated measures ANOVA examining percent of invest decisions by partner type, which revealed a strong main effect of partner, F(2,56) = 33.41, p < 0.001. Follow up paired t-tests on specific partner types revealed that individuals invested with FAIR > INDIFFERENT, t(28) = 5.08, p < 0.001, and INDIFFERENT > UNFAIR t(28) = 3.73, p = 0.001 (Figure 2a). We also provide a figure of average investment behavior over the 20 trials in which participants encountered each partner type, which shows that participants decoded partner reputation quickly, with differentiation between partner types established by the 6^th^ encounter with each partner type (Figure 2b).

### fMRI Results

**Figure 3:**
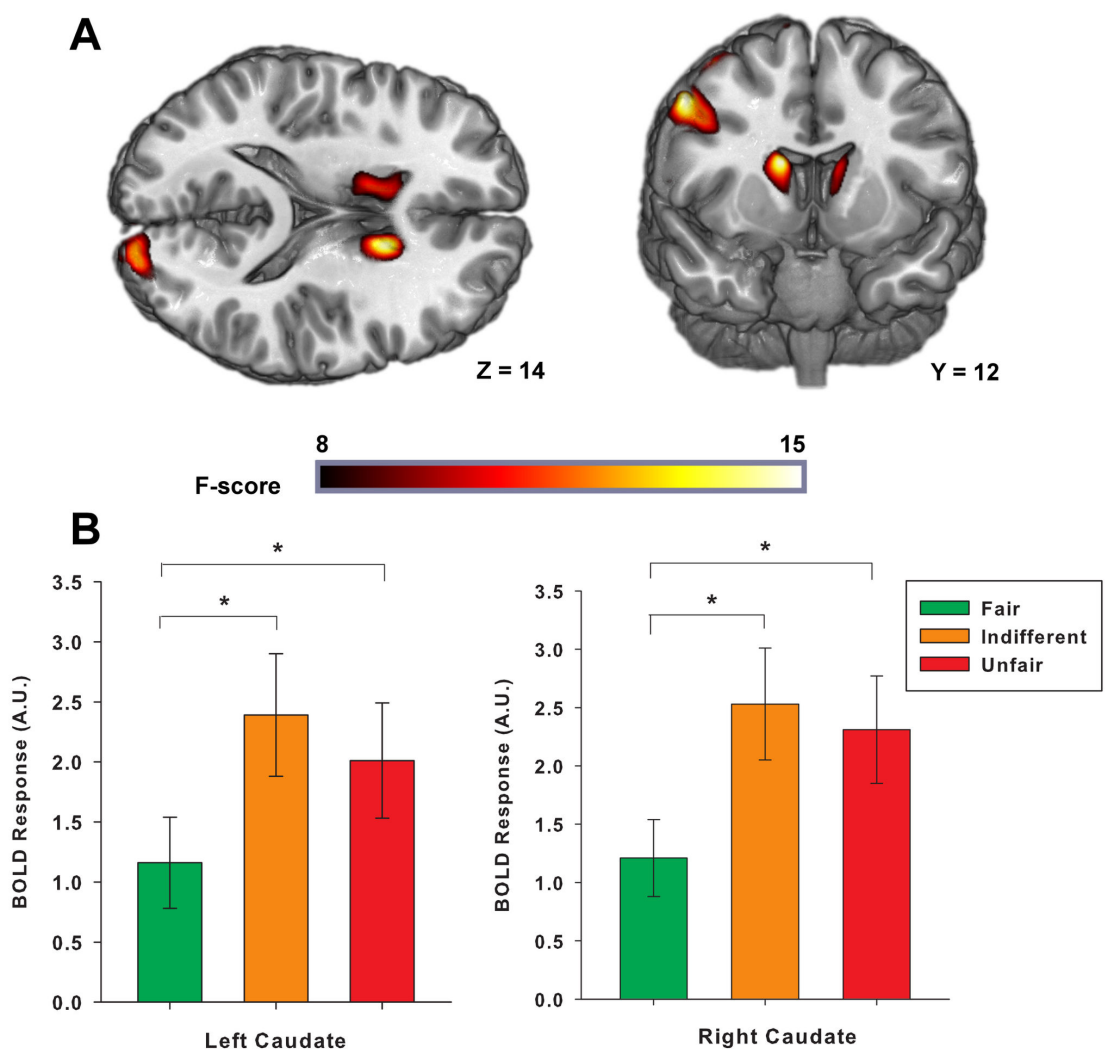
Caudate activity during decisions is bilaterally affected by reputation. A. Main effect of reputation on caudate activity displayed on a canonical T1 template (all activations whole brain p < 0.10, based on 3dClustSim correction [cluster size > 212, p < 0.001 uncorrected]). **B**. Both left and right caudate activity is increased to INDIFFERENT and UNFAIR partners relative to FAIR partners. Shown as parameter estimates extracted from 10mm spheres around areas of peak activity in left and right caudate (SEM), * p < 0.05.

**Figure 4:**
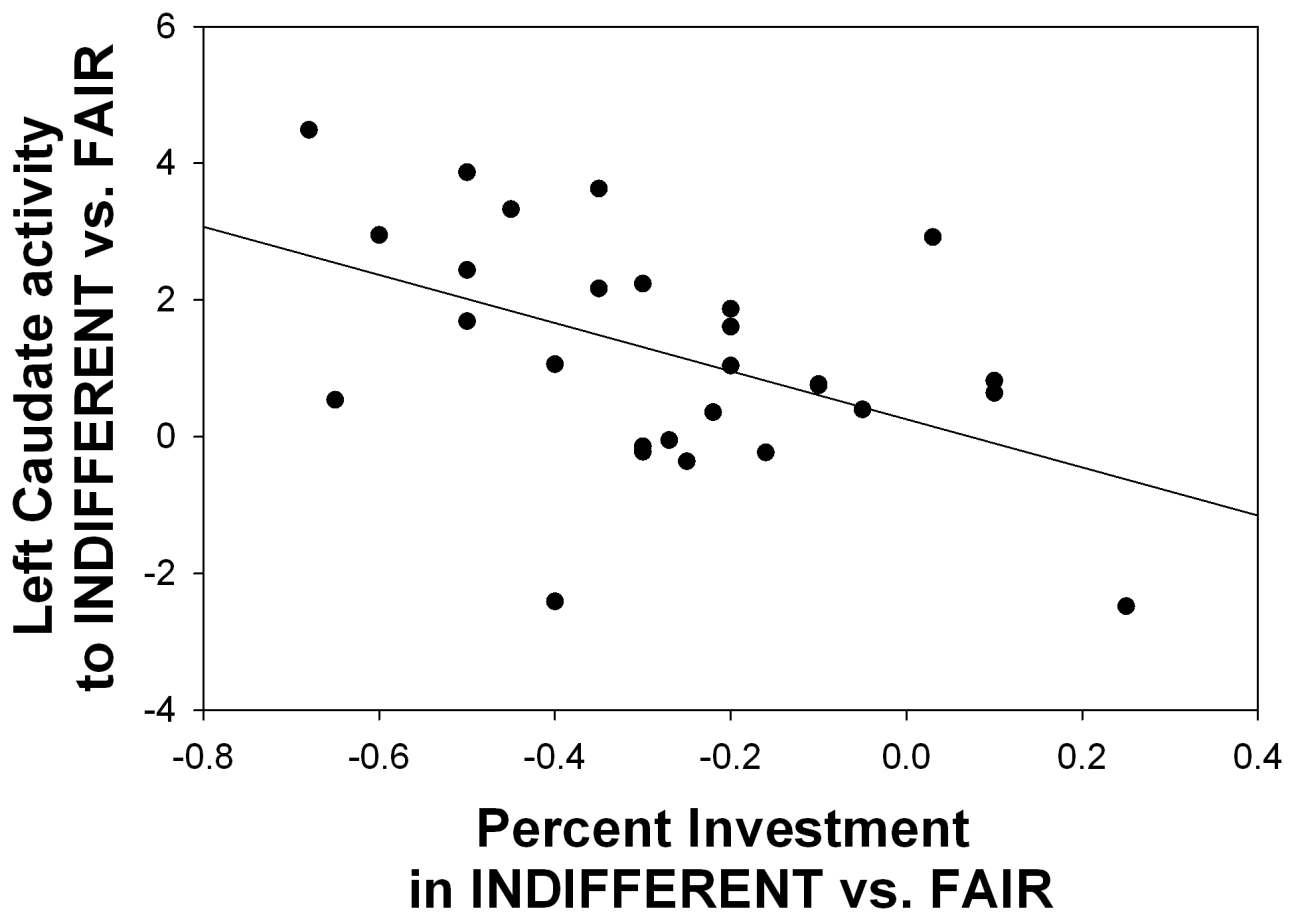
Higher left caudate activity to INDIFFERENT vs. FAIR partners predicted lower relative investment in INDIFFERENT vs. FAIR partners, r = -0.48, p = 0.01.

Of those areas designated *a-priori*, i.e. caudate, putamen (VS) and amygdala, only the caudate survived our significance threshold. The right caudate was significantly activated at p < 0.05 in both the ROI (right [14,12,16]:, Z = 15.17, p = 0.001 FWE-SVC) and whole brain analyses (see Table 1), while the left caudate was significantly activated in the ROI analysis (left: [-10, 10, 16], Z = 10.89, p = 0.01 FWE-SVC), and marginally significantly at p < 0.10 in the whole brain analysis (see Table 1). Follow up analyses of parameter estimates from 10mm spheres around these peaks indicated that both left and right caudate were more active to INDIFFERENT and UNFAIR partners, compared to FAIR partners (p < 0.01, corrected for four comparisons, Figure 3). There were no significant differences between INDIFFERENT and UNFAIR partners. Caudate activation was also not significantly moderated by the participant’s eventual KEEP vs. INVEST decision. We then examined whether individual differences in caudate activation were related to investment behavior. We constructed indices of relative caudate activation to INDIFFERENT and UNFAIR partners vs. FAIR partners by subtracting caudate activation to INDIFFERENT and UNFAIR partners from caudate activation to FAIR partners. We then constructed indices of relative tendency to invest in INDIFFERENT and UNFAIR partners vs. FAIR partners by subtracting investment rates for INDIFFERENT and UNFAIR partners from investment rates for FAIR partners. We removed one outlier with an unusually high rate of investment in INDIFFERENT vs. FAIR partners. Correcting for family-wise error across these four comparisons, we found stronger left caudate activation to INDIFFERNT relative to FAIR partners was significantly related to less investment in INDIFFERENT relative to FAIR partners; r(28) = -0.48, p = 0.01, see Figure 4. A similar trend existed for right caudate activity; r(28) = -0.35, p = 0.07. Relative caudate activation to UNFAIR partners was not significantly related to relative rates of investment in UNFAIR partners.

**Table 1:** Activations exhibiting main effect of reputation during the decision phase.

**Brain Region**	**Volume (Voxels)**	**F-Score**	**MNI coordinates**		
			x	y	z
**R Middle Temporal Gyrus**	785	21.88	60	-42	-12
**R Cerebellum**	538	16.52	20	-76	-18
**R Precentral Frontal Gyrus**	1021	15.61	52	8	42
**R Inferior Parietal Lobule**	2059	15.2	52	-44	52
**R Caudate**	338	15.17	14	12	16
**R Cuneus**	484	13.21	16	-96	12
**L Inferior Parietal Lobule**	433	11.81	-44	-50	46
L Caudate	250	10.89	-10	10	16

Areas in bold met whole-brain corrected significance at p < 0.05 (determined by 3DClustSim), Other areas marginally significant at p < 0.10 (determined by 3DClustSim)

Secondarily, to suggest other possible areas involved in reputation for future investigation and replication, we report all areas activated by the main effect of partner at p < 0.05 or p < 0.10 whole-brain corrected in Table 1. We extracted parameter estimates from 10mm spheres around these peaks for follow up analyses examining the direction of these effects, and whether these effect were moderated by eventual KEEP/INVEST decision. The area identified in the right middle temporal gyrus (60, -42, -1), similar to the caudate, was more active to INDIFFERENT and UNFAIR partners, compared to FAIR. There was no difference in activity between the INDIFFERENT and UNFAIR conditions, and no moderation of this effect by eventual decision. The right cerebellum (20, -76, -18) demonstrated a similar pattern of greater activity to INDIFFERENT and UNFAIR partners, but this activity was moderated by eventual investment decision such that increased activity to “bad” partners in the cerebellum was more evident when participants chose KEEP than when they chose INVEST; partner x choice F(2,44) = 6.23, p = 0.004. The right precentral gyrus (52,8,42) showed the same pattern as the cerebellum, with increased activity to “bad” partners that appeared primarily when the participant chose KEEP; partner x choice F(2,44) = 3.55, p = 0.04. The left and right inferior parietal lobules were both more active to INDIFFERENT and UNFAIR compared to FAIR, with no moderation of this effect by eventual investment decision. Finally, the right cuneus (16, -96, 12) displayed the most complex partner x choice interaction; F(2,44) = 8.35, p = 0.001. Here, when the participant chose to invest, activity was similar to FAIR and INDIFFERENT partners, but actually declined significantly to UNFAIR partners. In contrast, when the participant chose to keep, the cuneus demonstrated a similar pattern to our other areas, with increased activity to INDIFFERENT and UNFAIR partners compared to FAIR. This was also the only area reaching whole brain significance that activated differentially to INDIFFERENT vs. UNFAIR partners under any conditions.

## Discussion

We used event-related fMRI and a unique real-time reputation manipulation to examine which brain areas signal partner reputation during trust decisions. Of the three regions we hypothesized *a priori* would be sensitive to reputation, i.e. caudate, putamen (VS) and amygdala, only caudate was responsive to partner reputation during trust decisions. Caudate was more active bilaterally when the participant was faced with partners who reciprocated half of the time or less, relative to partners who reciprocated more than half of the time. Further, individual differences in caudate activity were related to biases in investment behavior in situations with the highest level of uncertainty, i.e. when facing partners who reciprocated only 50% of the time. Individuals who had relatively higher caudate activity to indifferent partners relative to fair partners invested relatively less in those partners compared to fair partners. Our findings show that caudate represents reputations formed in an ecologically valid manner through repeated experience, and suggests individual differences in the strength of this activity may correspond to tendency to trust in uncertain situations.

Our findings suggest a specific role of the caudate during decision-making is to signal the presence of “bad” or risky partners. Consistent with our findings, in two previous studies caudate activity was higher during the decision phase when participants were faced with a partner with a “bad” reputation acquired indirectly, specifically someone from a less-trusted race, or someone with a bad moral character [11,12]. Our results differ somewhat, as in these studies caudate activity was moderated by eventual decision, with differences between reputations appearing only during invest decisions. In contrast, our results suggest that the caudate signals whenever a “bad” partner appears, but does not reflect information about eventual decisions. This interaction between partner and decision may not have appeared in our study because there was insufficient power to detect it, or it may be that the effects of an indirect reputation manipulation on the caudate differ from those of a direct manipulation like the one used here. Our results also diverge from one study that manipulated reputation by having participants first play a simulated game of catch in which partners either included or excluded the participant [13]. That study failed to detect differential caudate activity to “bad” partners during trust decisions. However, it is difficult to disentangle what is motivating decision-making in such a paradigm, as impressions of trustworthiness are accompanied by additional variables such as feelings of exclusion. In sum, the balance of studies that have indirectly manipulated reputation are congruent with our findings using a direct reputation manipulation, that is, the caudate represents reputation by signaling the presence of “bad” partners during decision-making.

This role for the caudate in maintaining information about reputation is consistent with the non-social decision-making literature, in which the caudate is often identified with the “actor” in the actor-critic model of reinforcement learning, i.e. that portion of the learning system which maintains information about outcomes of given actions to facilitate good decision-making [21]. The design of the current study does not allow us to determine whether the signal we observed in the caudate is unique to bad social reputation, or whether similar patterns would be observed in response to non-social cues signaling likely punishment. Studies comparing social outcomes to monetary outcomes suggest overlapping circuitry, including the caudate, process both social and non-social rewards [22,23], but there has been no direct comparison of social vs. non-social cues of reward probability. However, there are some suggestions that our results are unique to social cues, as previous studies investigating anticipation of monetary rewards and punishment have typically found increased caudate activity to cues signaling both likely reward and likely punishment [24-26], rather than activation primarily to cues signaling likely punishment, as seen in the current study. Future studies directly comparing social and non-social cues will be needed to determine whether this caudate activity is part of a unique system for processing social cues, or is common to learning of all types of cues, including reputational ones.

Interestingly, previous studies indicate caudate activity is also evident during other phases of the trust game, but the direction of this activity is different than might be expected if caudate solely signals “bad” reputation or risk in all phases of trust. Specifically, caudate activity is higher in investors in response to cooperative outcomes [12,13]. Further, higher caudate activity in trustees when investor choice is revealed predicts that the trustee will subsequently cooperate with the investor [9,27]. Thus, our findings add to a literature indicating that the caudate is critical to decisions about trust, but also suggests caudate activity may play different roles during decision-making vs. outcome, or in initial trust decisions vs. decisions to reciprocate.

In the current study, caudate activity did not distinguish between indifferent partners who reciprocated 50% of the time and actively unfair partners who reciprocated less than half the time. We hypothesize that this may be because both indifferent and unfair partners are “bad” by standards of typical trust game play. Rates of reciprocity in iterated trust games are generally high in unconstrained participants, particularly in early rounds where impressions are initially formed [9,28]. Thus, the caudate may respond in a binary fashion to higher than expected levels of non-reciprocity or risk, rather than being linearly calibrated to reciprocity levels. Further examination with more finely differentiated levels of reciprocity will be necessary to examine this possibility.

Our preliminary findings relating individual differences in caudate activity to individual differences in willingness to invest support the idea that the caudate is also involved in dispositions to be trusting. This result must be treated cautiously, as our study was not designed to investigate individual differences, and the relationship only appeared in decisions involving indifferent, not unfair partners. However, it is consistent with previous findings. In a study examining trust for racial groups, higher caudate activity to an untrusted vs. trusted racial group correlated with lower investment in the untrusted vs. trusted group [11], similar to our findings with indifferent vs. fair partners. Further, exogenous administration of oxytocin increases repeated trust of indifferent partners by reducing caudate activity [10]. It may be that we only found a relationship between behavior and caudate activity in the indifferent condition because responses to indifferent partners were the most variable (ranging from identical to fair partners to identical to unfair partners), providing the greatest opportunity to observe a relationship.

We additionally reported on several unpredicted areas that reached whole brain significance on an exploratory basis, including the right middle temporal gyrus, right cerebellum, right precentral gyrus, left and right inferior parietal lobules and the right cuneus. Most of these areas demonstrated a pattern similar to that seen in the caudate, such that both “bad” partners increased activity, but there was no differentiation between unfair and indifferent partners. In the few areas where this effect was moderated by eventual investment decision, it appeared stronger when the participant eventually chose to keep the money. Some of these areas have previously been linked to social cognition, and are likely candidates for further exploration and replication. In particular, it has been suggested that the middle temporal gyrus may be involved in “mentalizing” and attribution of intention to others, and specifically with integrating episodic memory into representations of the intentions of others [29,30]. This would clearly be a key component of establishing and updating reputations, perhaps especially so when confronted with “bad” actors whose intentions are more uncertain or non-normative. The other areas identified in our hypothesis-free analysis have less obvious connections to reputation. However, the inferior parietal lobules and precentral area have been implicated in empathy and social cognition via the “mirror neuron” system [31]. This system consists of motor areas that activate both when individuals are performing and when they are observing an action, and it is hypothesized to be a key basis for understanding the mental and physical states of others. It is less clear how this system may be involved in a comparatively motor-free “mentalizing” task such as discerning the intention of a partner on an investment task, but it may be that some form of embodied cognition is involved even in this primarily non-physical task [32]. Finally, the cerebellum and cuneus are comparatively less implicated in social cognition, being primarily associated with motor control and visual attention respectively, but it is possible that these systems are more fully engaged when confronted with “bad” actors than otherwise.

These findings should be considered in the context of the following limitations. As noted above, we may have lacked power to detect the interaction between reputation and eventual decision evident in other studies with larger sample sizes, e.g. [11]. Further, it is surprising that our other *a priori* regions of interest were not activated by reputation. We previously reported a signal for reputation in VS during outcomes when trustee behavior is revealed [14], so it may be that VS is more relevant to the outcome phase of trust interactions. However, this hypothesis would be at odds with the “prediction error” literature, which suggests VS activity is higher during cues signaling reward [33]. In addition, previous studies have actually found higher VS activity to “bad” reputation during trust decisions, which is both inconsistent with prediction error theory, and at odds with our results [12]. The role of this area in trust decisions will be important to clarify in future studies. Regarding amygdala, it is possible the amygdala is more relevant to trust when facial expressions are visible, e.g. [34], rather than obscured as they were here, although we cannot investigate this possibility in the current study. Another primary limitation was restriction of our ROI based examination to three key regions of interest. Although we selected regions with the most previous evidence for representing reputation, there are many other areas implicated in trust, including insula [35],, anterior cingulate cortex [27], the septal area [8], and anterior medial prefrontal cortex [18], that we lacked power to examine. As noted above, this study was not designed to examine individual differences in investment behavior. Our finding that individual differences in caudate activity may relate to biases in trust behavior is intriguing, but will require confirmation. Finally, our hypothesis-free whole brain analysis results are presented primarily for further replication and confirmation, as although some of the areas identified have an established connection to social cognition, others do not. The possibility that these are false positives will need to be examined in future studies.

In sum, this study is the first to examine how the brain represents reputations acquired through repeated interactions during trust decisions. We demonstrate that the caudate signals “bad” reputations during trust decisions, and further, that the strength of this signal may relate to individual biases in decision-making. Given known relationships between ability to build trust and the well-being of individuals, economies and nations, this study provides important information about the brain mechanisms underlying the critical process of using reputation information to guide trust decisions.
